# The Association between Long Non-Coding RNAs and Alzheimer’s Disease

**DOI:** 10.3390/brainsci14080818

**Published:** 2024-08-15

**Authors:** Carson M. Black, Anneliesse A. Braden, Samia Nasim, Manish Tripathi, Jianfeng Xiao, Mohammad Moshahid Khan

**Affiliations:** 1 Departments of Neurology, College of Medicine, University of Tennessee Health Science Center, Memphis, TN 38163, USA; carson.m.black@gmail.com (C.M.B.); jxiao@uthsc.edu (J.X.); 2 Neuroscience Institute, University of Tennessee Health Science Center, Memphis, TN 38163, USA; 3 Departments of Ophthalmology, Hamilton Eye Institute, University of Tennessee Health Science Center, Memphis, TN 38163, USA; snasim1@uthsc.edu; 4 Medicine and Oncology, University of Texas Rio Grande Valley, McAllen, TX 78504, USA; manish.tripathi@utrgv.edu; 5 Division of Regenerative and Rehabilitation Sciences, Department of Physical Therapy, Center for Muscle, Metabolism and Neuropathology, College of Health Professions, University of Tennessee Health Science Center, Memphis, TN 38163, USA

**Keywords:** long non-coding RNAs, neurodegeneration, Alzheimer’s disease, biomarker, gender, therapy

## Abstract

Neurodegeneration occurs naturally as humans age, but the presence of additional pathogenic mechanisms yields harmful and consequential effects on the brain. Alzheimer’s disease (AD), the most common form of dementia, is a composite of such factors. Despite extensive research to identify the exact causes of AD, therapeutic approaches for treating the disease continue to be ineffective, indicating important gaps in our understanding of disease mechanisms. Long non-coding RNAs (lncRNAs) are an endogenous class of regulatory RNA transcripts longer than 200 nucleotides, involved in various regulatory networks, whose dysregulation is evident in several neural and extraneural diseases. LncRNAs are ubiquitously expressed across all tissues with a wide range of functions, including controlling cell differentiation and development, responding to environmental stimuli, and other physiological processes. Several lncRNAs have been identified as potential contributors in worsening neurodegeneration due to altered regulation during abnormal pathological conditions. Within neurological disease, lncRNAs are prime candidates for use as biomarkers and pharmacological targets. Gender-associated lncRNA expression is altered in a gender-dependent manner for AD, suggesting more research needs to be focused on this relationship. Overall, research on lncRNAs and their connection to neurodegenerative disease is growing exponentially, as commercial enterprises are already designing and employing RNA therapeutics. In this review we offer a comprehensive overview of the current state of knowledge on the role of lncRNAs in AD and discuss the potential implications of lncRNA as potential therapeutic targets and diagnostic biomarkers in patients with Alzheimer’s disease.

## 1. Introduction

Neurodegenerative diseases are a broad classification of a heterogeneous group of disorders characterized by the progressive degradation of neurological structure and function in the central nervous system (CNS) and peripheral nervous system. Ultimately, the degradation of the impacted brain regions result in consequent decline in bodily function. Herein, the article focuses on one of the most common neurodegenerative diseases and the most common form of dementia, Alzheimer’s disease (AD).

Alzheimer’s disease presents with memory loss, confusion, learning deficits, alterations in smell and taste, and gait abnormalities. Risk factors for AD include cardiovascular diseases, age, high cholesterol, diabetes, positive family history, traumatic brain injury, and the presence of variant 4 of apolipoprotein E (APOE4), amongst other factors yet to be described [1,2]. AD is categorized into two subtypes, sporadic or late-onset AD (LOAD) and familial or early-onset AD (EOAD). The most prevalent is LOAD, accounting for up to 90% of all AD cases [3]. Although less frequent, EOAD often occurs between the ages of 30 and 50 years and may be more severe or progressive than sporadic cases [2]. The presence of AD-associated pathology can lead to varying degrees of extracellular buildup of toxic amyloid beta (Aβ) peptides and an accumulation of neurofibrillary tangles (NFTs) in multiple brain regions [1]. AD incidence is twice that in females as in males [4]. Despite decades of research, there are no effective therapies to slow or halt the progression of cognitive dysfunction associated with AD. This highlights the need to advance our knowledge of the molecular mechanisms underlying the process of neurodegeneration and to pinpoint effective therapeutic targets. While protein-coding genes have largely been studied in neurodegenerative diseases, growing evidence connects long non-coding RNAs (lncRNAs) with AD pathogenesis [5,6,7,8,9].

**Figure 1:**
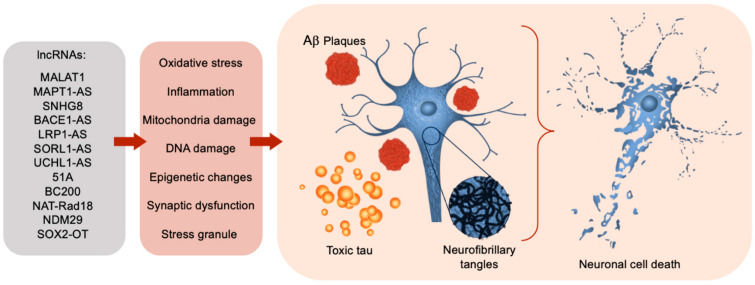
LncRNAs functional significance in AD progression and pathology. The far-left lists examples of lncRNAs dysregulated in Alzheimer’s disease pathology. Following the red arrow on the right, these lncRNAs lead to some of the biological functions implicated in AD listed in the red box. The physiological and structural outcomes are next. Aβ plaques, imaged in a dark red, surround a neuron affected with neurofibrillary tangles (NFTs). The molecular NFT structure is examined with closer detail to the right of the neuronal cell body. The tangles are made of unstable filaments of hyperphosphorylated microtubule-associated tau protein (MAPT). The toxic tau species is illustrated to the left of the neuronal cell body. The aggregation of abnormal proteins further leads to neurodegeneration and ultimately cognitive deficits.

Long non-coding RNAs fundamentally resemble messenger RNA (mRNA) but do not encode proteins [8]. Their expression is often limited to particular cells or developmental stages [10]. LncRNAs may function as scaffolding, decoys, miRNA sequestrators, and histone modifiers and may be integral in transcriptional processing and silencing [11]. LncRNAs contribute to the regulation of several physiological and pathophysiological processes [6,7,8,9,10]. By communicating DNA, RNA, and proteins, lncRNAs regulate chromatin remodeling; transcription and post-transcription by recruiting or limiting transcription factors; epigenetic functions and alternative mRNA splicing; stability; and translation. All this ultimately affects gene expression in diverse biological and pathophysiological conditions [5,6,7,8,9,12]. LncRNAs are also involved in organelle and nuclear condensate genesis and regulation [12]. In addition to the various physiological functions, lncRNAs are connected to a broad range of pathological phenotypes due to changes in transcript levels between normal and disease conditions [13,14,15]. LncRNAs have been shown to play a critical role in major causal agents in AD pathogenesis including tau phosphorylation, synaptic plasticity regulation, and APP processing [16,17,18,19] (Figure 1). LncRNAs also interact with miRNAs and through this influence AD-related neuropathology [5]. Furthermore, the expression of several lncRNAs have been detected in plasma and CSF samples from AD patients, indicating lncRNAs could function as a likely biomarker in the diagnosis and progression of AD [5,20,21]. These findings deliver proof-of-concept evidence and support that lncRNAs, similarly to protein-coding genes, play crucial regulatory roles in diverse neurobiological processes in AD. Thus, understanding how lncRNAs regulate neuronal function may allow for the development of diagnostic markers as well as effective therapies.

Research articles related to long non-coding RNAs and their interactions with neurodegeneration have increased in number in recent years. As of 2010, there were nine total articles published on PubMed using the keywords, “lncRNA, neurodegeneration”. As of July 2024, there are 206 articles using the same keywords. The field is growing rapidly. This review article summarizes emerging research detailing the role of lncRNAs in AD and discuss the implications of lncRNA as a potential therapeutic target and diagnostic biomarker in AD. Additionally, we discussed the effect of gender on lncRNA dysregulation and AD contributions.

### 1.1. Significance of lncRNAs

Seventy-six percent to ninety-seven percent of the human genome encodes for RNA that is not translated into proteins, known as non-coding RNA [22]. Long non-coding RNAs are a relatively new endogenous class of regulatory RNA transcripts longer than 200 nucleotides. They lack an open reading frame and do not code for a protein of particular interest; they instead serve intrinsically as RNA molecules [10]. They can directly interact with DNA and associate with enhancers or promotors to activate or suppress function. While many lncRNAs have been discovered, only some have been functionally described [22]. The functions, mechanisms, and locales of lncRNAs vary widely, but several have been identified, characterized, and implicated in neurodegenerative pathologies including neuronal aging, cognitive decline, and diseases [9,23]. LncRNAs keep dynamic and tight control of CNS development and can significantly influence many other biological processes. The GENCODE project estimated the existence of 18,000 human lncRNAs [10]. Since then, the NONCODE database has annotated 96,411 human lncRNA genes estimated to produce 173,112 lncRNA transcripts [22]. They considerably affect various signaling pathways via gene expression regulation at the transcriptional, post-transcriptional, and translational levels. LncRNAs have a considerable role in regulating cell proliferation and differentiation during development [3,24,25]. Riva et al. stated that lncRNA dysregulation may influence the processing, stability, protein translation, and localization of several different target transcripts [3]. LncRNAs show elevated tissue specificity and dynamic expression throughout development [22]. Research efforts have significantly increased since the discovery that lncRNAs are intimately involved in disease states due to the direct and indirect functions and levels. Specific diseases are modulated by the quantity of lncRNAs as well as the quantity of molecules subject to the downstream effects of lncRNA effects, resulting in significant dysregulation of biological pathways. Although much work remains, several lncRNAs are already characterized and being used as biomarkers or therapy targets in some cases of cancer, cardiovascular abnormalities, and diabetes mellitus [2,26,27,28]. LncRNAs have been shown to impact host-microbe interactions within the gut, and thus may affect several human neural and extraneural diseases [29,30]. LncRNAs are also involved in the biological process of viruses [31,32]. Most notably, lncRNAs regulate the biological process involved with SARS-CoV2, making them a valuable target [33]. Hopefully, with further research focused on the specific roles of lncRNAs, a myriad of novel clinical and subclinical uses can improve treatments or develop cures that have otherwise been elusive.

### 1.2. Biological Functions of LncRNAs in Health and Disease

Several lncRNAs have been identified as potential contributors in worsening neurodegeneration due to altered regulation during abnormal neurological conditions. In comparison with healthy populations, Chen et al. and Yang et al. found that at least 77 lncRNAs are severely downregulated, and 238 lncRNAs are significantly upregulated in AD patient brains [34,35]. Therefore, it is of utmost importance to fully understand their molecular mechanisms and interactions to advance neurodegenerative disease research. By exploring lncRNA mechanisms more completely and precisely, it will be possible to determine how lncRNAs function and regulate key pathobiological mechanisms underlying disease conditions. Eventually, this will allow the development of effective therapeutic strategies for the treatment of several neural and extraneural diseases.

**Figure 2:**
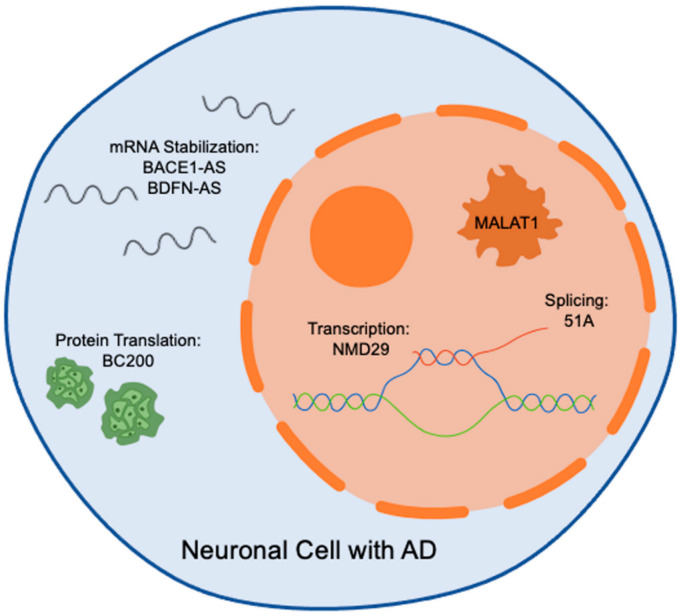
LncRNA roles within neurons in AD pathology. The cell body of a neuron is illustrated by the encapsulating blue circle, in which, the nucleus is represented by the dashed orange circle. Within the nucleus, transcription and splicing take place. The blue and green lines represent the coding and template DNA strands. Transcription is represented by the addition of red RNA created from the blue template DNA. The newly made precursor messenger RNA is then spliced to create mature mRNA. Examples of dysregulated lncRNAs relevant are MALAT1, NMD29, and 51A. Moving to the cytoplasm, examples of significant lncRNAs abnormally represented in AD are BACE1-AS, BDFN-AS, and BC200. The pertinent cytoplasmic processes include mRNA stabilization and protein translation.

LncRNAs are expressed across all tissues, including those in the CNS, with precisely regulated spatial and age-dependent patterns [36]. LncRNAs are often transcribed by RNA polymerase II or III, induced by promoters, are frequently polyadenylated, and may participate in alternative splicing [35,37]. According to Wan et al. and the GENCODE classification system, lncRNAs are most commonly classified by their length (i.e., size) and by their localization and function [37,38]. This organizational method promotes a more efficient understanding and facilitates a prediction of function because their complex mechanisms are closely tied to specific locations and by the entities they interact with; classification specifications and annotations have been detailed by Wan et al. [37] (Figure 2). LncRNAs amass in many subcellular compartments and organelles with a significant inclination towards the nucleus [13,39]. In the nucleus, nucleoli, chromatin speckles, and paraspeckles are the primary sites of location. In cytoplasm, lncRNAs have been found in mitochondria, exosomes, extracellular membrane, and ribosomes [13]. Several lncRNAs can be found in more than one organelle or compartment. The location of lncRNA’s transcription within the genome can change the importance of their regulatory capabilities.

LncRNAs regulate a wide range of functions, including controlling cell development, responding to environmental stimuli, and either directly or indirectly regulating the cellular homeostasis through their action on RNA-binding proteins (RBPs), RNA, DNA, proteins, other ncRNAs, or chromatin modifiers [1,40,41,42]. LncRNAs regulate gene expression at the epigenetic, transcriptional, post-transcriptional, translational, and post-translational levels by diverse mechanisms that are not yet fully understood [12,43,44]. LncRNAs can modulate the expression of neighboring genes via cis regulation or distant genes through trans-acting regulation. Furthermore, lncRNAs have an intimate relationship with protein-encoding genes, often overlapping them or occupying nearby intronic regions. This relationship allows lncRNAs to significantly influence gene transcription and protein turnover, as well as histone modification and chromatin organization in the nucleus [35,45]. In addition, lncRNAs play role in transcription, protein translation, and mRNA decay [46]. For instance, lncRNAs may interact with histone-modified complexes or enzymes or transcription factors to activate or silence gene transcription [44]. LncRNAs can act as precursors of miRNAs and siRNAs in addition to their interaction with miRNAs to regulate target gene expression in several biological processes [12,44]. The multiple gene regulatory activities of lncRNAs ultimately affect diverse aspects of physiology, from neuronal growth, synaptic dysregulation, and plasticity to key roles in several human neural and extraneural pathologies. LncRNA sequences are less conserved and contain fewer exons than their protein-encoding counterparts making them more versatile and more capable of responding to environmental changes such as inflammation, injury, and aging [47]. LncRNAs have been implicated in the preservation of neurons and other nervous system architecture during the degradation of normal aging. Although their levels seem to fluctuate during aging, their interventional modulation may be key to the preservation of important neurological interactions, structure, plasticity, learning, and memory, and to the curtailment or reversal of calamitous neurodegenerative processes [23]. Emerging studies have suggested that lncRNAs are implicated in Aβ processing, aberrant Tau deposition, oxidative stress, mitochondrial dysfunction, synaptic impairment, DNA damage response, epigenetic dysfunction, and immune response, causal agents of AD pathobiology (Figure 1) [5,13,48,49].

### 1.3. LncRNAs Dysregulation in Alzheimer’s Disease

Memory deficits are one of the earlier overt symptoms observed in AD patients. Apart from its cardinal cognitive symptoms, AD is associated with a heterogenous spectrum of non-cognitive symptoms that contribute significantly to the overall disease burden. Despite gaining considerable knowledge over the last several decades, the exact mechanism of AD remains unclear. There is growing evidence that several lncRNAs are dysregulated in AD or AD-related dementia, and directly or indirectly regulate the key features of AD including Aβ deposition, aberrant tau formation, oxidative stress, neuroinflammation, and neuronal death (Figure 1) [5,6,7,8,9]. Examples of lncRNAs implicated in the pathogenesis of AD are listed in Table 1. The major determinant of Aβ formation is beta-site amyloid precursor protein cleaving enzyme 1 (BACE1). Its antisense transcript (BACE1-AS) has often been observed and reported to positively regulate BACE1 protein content and promote synthesis of harmful Aβ42 [3,18]. This occurs by preventing miRNA 485-5p from binding and by increasing the stability of BACE1 mRNA [3]. BACE1-AS expression can be prompted by a stressor in the cell, including Aβ itself. In multiple studies, increased expression of BACE1-AS has been found in brains of AD patients, indicating the mechanism exacerbates itself progressively once initiated [3,20,23,50].

Nuclear paraspeckle assembly transcript 1 (NEAT1) is a widely studied lncRNA and has been shown to participate in several biological and pathological conditions. Multiple studies have indicated increased expression of NEAT1 lncRNA in AD [56]. The regulatory role of lncRNA NEAT1 is further supported by studies that show NEAT1 mediates neuronal damage following Aβ treatment, while inhibition of lncRNA NEAT1 limits Aβ production and ameliorates cognitive deficits in APP/PS1 mice [57,58]. BC200 lncRNA is overexpressed in patients with AD localized entirely to the brain, therefore minimizing its use as a biomarker in living patients but opening the possibility of use as a pharmacological therapeutic target. Another viable target for pharmacologic intervention is BDNF-AS. Enhanced BDNF-AS levels are positively correlated with cognitive deficits in human and AD mouse models. Furthermore, experimental studies suggest that BDNF-AS induction triggers BACE1 overexpression through the regulation of miRNAs, ultimately promoting neurotoxicity [5,78]. LncRNA 51A overlapping with sortilin-related receptor 1 was also revealed to affect Aβ formation and upregulation in AD [65,66,73]. The lncRNA 17A is a 159-nucleotide antisense transcript, localizes into intron 3 of the human G-protein coupled receptor gene, and regulates autophagy and apoptosis by targeting GABA-B receptor 2 [79]. LncRNA17A expression is higher in the cerebral cortex of AD patients [79]. A recent study by Yue et al. demonstrated that through the alteration of miR-124, AD-related BACE1 levels declined when lncRNA XIST was silenced, suggesting this lncRNA as a potential therapeutic target for AD [80]. Sox2OT promotes neurogenesis and neuronal differentiation but is downregulated in AD [66,72]. Furthermore, Sox2ot and 1810014B01Rik are described as potential biomarkers in all stages of neurodegenerative diseases [24,73]. TIA1, an RNA-binding protein correlated with tau and stress granules, is influenced by the lncRNA SNHG8. When there is overexpression of toxic tau, SNGH8 expression is reduced, promoting the formation of stress granules. SNHG8 is downregulated in AD brains resulting in increased stress granule formation [61]. Another aspect involved in AD pathogenesis is the impact of antisense (AS) lncRNAs. AS lncRNAs demonstrate a vital importance in the regulation or dysregulation of abnormal protein aggregates (Figure 2). Nuclear-expressed AS lncRNAs bind chromatin-regulating proteins, thus controlling when certain coding genes are transcribed [23]. The buildup of NFTs in AD is associated with an abnormal decrease of the lncRNA MAPT1-AS. This lncRNA aids in relieving aberrant tau within the neuron [59]. When absent, toxic tau builds up and creates NFTs, a hallmark pathology in AD [60]. NAT-Rad18, a natural antisense transcript of Rad18, encodes a series of the DNA repair protein and participates in the regulation of cell apoptosis. In response to Aβ treatment, lncRNA NAT-Rad18 is up-regulated in cortical neurons and increases the susceptibility to neuronal apoptosis [25,70].

MALAT1 (metastasis-associated lung adenocarcinoma transcript 1) is a highly abundant and evolutionary conserved lncRNA with many roles within the body [10]. Recent reports in experimental models of AD provide robust evidence and support for the potential roles of lncRNA MALAT1 in AD pathogenesis [17,51,52]. A recent report in mouse models of AD indicated enhanced neurite outgrowth, reduction in proinflammatory cytokines, and decreased neuronal apoptosis with lncRNA MALAT1 overexpression, and vice versa with lncRNA MALAT1 knockdown [17]. Reported by Bernard and coworkers, the suppression of lncRNA MALAT1 reduced synaptic density and increased presynaptic bouton density on dendrites by overexpression [51]. Consistently, lncRNA MALAT1 preserves survival and neurite outgrowth of neuro-2a neuroblastoma cells, and a knockdown model resulted in neurite outgrowth deficits and elevated cell death [53]. In a cellular model of AD, Yang et al. have reported that silencing of MALAT1 promotes apoptosis, whereas overexpression inhibits apoptosis [54]. In cancer models, MALAT1 lncRNA is upregulated, as it promotes cell growth and reproduction. To promote downregulation of MALAT1, and therefore rescue the cancerous phenotype, a 3′ termini triple helix structure responsible for stabilization and preventing exonuclease degradation is targeted and altered causing physical destabilization of MALAT1 [10,81]. LncRNA MALAT1 is downregulated in AD. The very same mechanism of destabilization of MALAT1 occurring in cancers may be active in AD presentation. Selectively promoting the upregulation of MALAT1 in neurons for a patient with AD could increase neurite outgrowth, aid in apoptosis inhibition and neuroinflammation regulation. Dysregulated lncRNAs in AD may have the capability to be targeted and rescued with the proper investigations.

### 1.4. LncRNAs as Potential Therapeutic Targets in Alzheimer’s Disease

Deep-transcriptome sequencing and genome-wide analyses have identified several dysregulated lncRNAs in the brains of AD patients and in mouse models of AD. Thus, understanding how lncRNAs regulate neuronal functions and fate in the brain may allow for the development of effective neurodegenerative disease therapies. BACE1 play important role in the overt production of the Aβ peptide implicated in AD pathogenesis. LncRNA BACE1-AS has been shown to increase the stability of BACE1 mRNA, and increased expression of LncRNA BACE1-AS is documented in the brain and plasma of AD patients [62]. Zhang et al. silenced BACE1-AS by short interference of RNA and rescued memory and learning behaviors in SAMP8 mice. They found increased primary hippocampal proliferation in vitro through BACE1 inhibition, APP production, and tau protein phosphorylation [63]. Therefore, targeting BACE1-AS seems to be a positive pathway forward in AD treatments. Other characterized lncRNAs whose pharmacological downregulation may be beneficial in AD include: 17A, 51A, NDM29, and NAT-Rad18 [2,24,35,70,82]. For instance, overexpression of lncRNA 17A amplifies the Aβ42-to-Aβ40 ratio and promotes apoptosis, where as its inhibition suppresses cell apoptosis and reduces the Aβ42-to-Aβ40 ratio in cultured neuronal cells [83], suggesting regulation of lncRNA 17A may provide effective therapeutic value. Similarly, NDM29 expression is higher in AD brains, and overexpression of NDM29 promotes APP synthesis, leading to the increase of Aβ formation in culture systems [71]. This finding suggests that modulation of NDM29 may control Aβ production, thus represent a potential therapeutic target in AD. A recent study by Lin et al. showed that lncRNA LINC00472 regulates ferroptosis signaling in AD by modulating iron accumulation in neuronal cells [84], suggesting regulation of lncRNA LINC00472 may have significant therapeutic potential in the treatment of AD. Similarly, lncRNA Maternally Expressed 3 (MEG3) was found to be upregulated in human neurons exposed to Aβ as well as in AD patient brains [85]. Interestingly, reduction of MEG3 protects neuronal cell loss in AD human neurons, suggesting a therapeutic potential of MEG3 in AD. The lncRNA small nucleolar RNA host gene 1 (SNHG1) has been shown to regulate several pathobiological mechanisms including neuroinflammation, DNA methylation, and apoptosis in neurodegenerative diseases including AD [74,75]. Enhanced expression of lncRNAs SNHG1 were observed in a cellular model of AD [76], and downregulation of its expression by resveratrol attenuates Aβ-induced neurotoxicity [77]. Thus, targeting SNHG1 may provide therapeutic benefits in AD. Recent studies demonstrated the role of lncRNA ANRIL in several vascular diseases such as coronary artery disease and atherosclerosis, diabetes, and dementia [86,87]. For instance, Zhou and coworkers showed that knockdown of lncRNA ANRIL suppressed cell apoptosis and inflammation while promoting neurite outgrowth in a cellular model of AD [88]. Given that vascular diseases increase the risk of AD onset and progression, it is reasonable to infer that targeting ANRIL can positively influence vascular health and reduce AD risk.

LncRNA LRP1-AS is upregulated in the brains of AD patients [64]. In effect, LRP1 is responsible for synaptic activity maintenance, dendritic and neuronal structure, as well as influencing Aβ agglomeration. LRP-AS binds HMGB2, a transcriptional activator, which blocks transcription and lowers LRP1’s expression level [64]. SORL1-AS is another upregulated lncRNA in AD, and it is responsible for the creation of deleterious splicing isoforms of SORL1, which have been shown to increase levels of Aβ [23]. In the nucleus of dopaminergic neurons, the lncRNA ubiquitin carboxy-terminal hydrolase L1 (UCHL1) antisense transcript causes increased UCHL1. This then ubiquitinates Aβ protein, thereby promoting plaque deconstruction and improving AD symptoms [24]. Thus, therapeutic modulation of these lncRNAs via pharmacological targeting could provide alleviation of symptoms and potential halting of disease progression. Fernandes et al. describe another subset of AS lncRNAs, those with cytoplasmic function; they either stabilize or destabilize gene transcripts with important roles in neurodegenerative diseases and are also important for the maintenance of normal cognitive function. For example, PINK1-AS stabilizes the PINK1 transcript; the PINK1 gene is responsible for protecting cells against oxidative stress-induced apoptosis via mitochondria. GDNF-AS stimulates the degradation of the GDNF transcript; this gene regulates neurite outgrowth, thus neuronal cell survival. EPHB2-AS promotes decreased the ephrin B2 receptor (EPHB2); a gene involved in synaptic plasticity and synaptogenesis. KCNA2-AS represses expression of the KCNA2 transcript; this gene causes a gain or loss of potassium flow in ion channels between neurons, thereby affecting neurotransmitter release and neuronal excitability [23]. Many differentially expressed transcripts are also involved in processes such as synaptic transmission, cholinergic regulation, differentiation of CNS neurons, ligand receptor interactions, G protein-coupled receptor (GPCR) interactions, axon positioning, mTOR signaling, MAPK signaling, PI3K-Akt signaling, glial cell processing, the renin-angiotensin-aldosterone system, and more [1]. The mechanisms of many other lncRNAs and their similarly acting antisense counterparts are well established, thus validating the suggestion that intentional manipulation could serve as the first-line, standard target in controlling the pathological processes they modulate. Certainly, sensitive and specific RNA processing techniques could be developed, generalized, and distributed based on a panel of lncRNAs and diseases being investigated. Individual RNA analysis could include reverse transcription and PCR analysis, and RNA panels could be developed using RNA sequencing, microarrays, or nanostrings [1]. The potential of RNA technologies in the treatment of AD is immense, but more work needs to be done.

### 1.5. Gender Specific lncRNAs in AD

The pathogenesis of AD is influenced by numerous variables evidenced most drastically by the incidence of AD occurring twice as often in females as in males [18,89]. There is stronger clinical correlation of AD brain pathologies (e.g., NFTs, neuritic plaques, and diffuse plaques) in female AD patients compared to male AD patients, underlining the significant need to examine the interplay of gender-associated factors in AD. Moreover, the overexpression of corticotropin-releasing factor in females is linked to the formation of NFTs via increased tau phosphorylation [18,89]. In 2019 Cao et al. explored the relationship of gender-associated lncRNAs and AD in substantial depth (*n* = 214). They found that healthy female brains have 37 differentially expressed lncRNAs compared to healthy male brains. Among the gender-associated lncRNAs found, 13 were significantly dysregulated in female and male AD brains compared to the healthy groups [18]. In a 2-way analysis of variance between gender and AD, they demonstrated that AD affects the expression of gender-associated lncRNAs in a gender-dependent way, providing more evidence of a gap in knowledge [18]. Exploring the role of lncRNAs in the relationship between AD incidence and gender may provide valuable insight into the twofold disparity women experience with AD.

### 1.6. LncRNA Therapy

Commercial enterprises are already designing and employing therapeutics such as ablation techniques and oligonucleotides to treat neurodegenerative diseases [24,82,90]. As of 2022, the total RNA medical product market capitalization surpassed $0.1 Trillion, solidifying a place among the most rapidly growing segments of modern medicine [91]. As of 2023, there were nearly 2000 RNA products in clinical and pre-clinical development [46], divided by RNA class as follows: 1013 Oligonucleotides, 390 gRNA-mediated gene editing, 375 mRNA, 97 RNA-targeted small molecules, and 46 circRNA [91].

The innate abilities of lncRNAs contribute to their potential as a valuable therapeutic or pharmaceutical target [10]. Unfortunately, their size and complex structure can instigate an immune response from the body. However, if the functionally relevant region is isolated and removed, the creation of artificial, smaller lncRNAs mimicking the effects of the full-sized lncRNA is possible [10]. Synthetic lncRNA can be combined with programmable synthetic RNA devices to fine-tune expression or direct subcellular localization [10]. In particular, antisense oligonucleotides (ASOs) targeting lncRNAs are a viable and specific option for treatment, although challenges such as delivery through the blood-brain barrier (BBB), undesirable bioavailability, and subjection to nuclease destruction are relevant [2]. Exosome nanovesicles (EVs) could provide protection via a protective shell for ASOs during delivery [2]. The most relevant challenge companies are facing involves actual drug uptake by the cell. Once permeating the BBB, the RNA drug typically enters the cell via endocytosis mechanisms, localizes to the cytoplasm or nucleus, and then exits the endosome. This is no easy feat, as the drug may remain trapped in the endosome it used to enter the cell, contributing to an even lower drug uptake [10]. The development of antagoNATs, natural antisense transcript antagonists targeting lncRNAs, could be a possible selective and individualized treatment for neurodegenerative diseases [37]. AntagoNATs may offer solutions to drug uptake challenges. In mice, they were applied via minimally invasive nasaldepot (MIND), resulting in 40% efficacy compared to riskier invasive methods. This technique directs drug delivery to the olfactory submucosal space, a much safer application method with high-clinical utility [81]. Using the natural abilities of lncRNAs to an advantage will further AD research and help more patients in the coming decade.

### 1.7. Diagnostic Potential of LncRNAs in AD

An early diagnosis of AD and early interventions are important and can stop the conversion of clinically asymptomatic to severe AD conditions. Currently, a diagnosis of AD relies on brain imaging and a fluid biopsy of Aβ and tau proteins; however, these generally predict the progression of cognitive deficits after they appear in the clinic. Therefore, there is an unmet medical need to identify biomarkers for early diagnosis of AD in more easily accessible specimens. Due to their specific expression, stability within the body, circulating nature in bodily fluids, and the direct relationship between altered expression and disease severity, lncRNAs present as a strong choice for noninvasive screening options [2]. For instance, studies have shown that plasma levels of lncRNA BACE1 were significantly high in AD patients as compared to healthy controls and this increased plasma expression level lncRNA BACE1 levels is positively correlated with severity of cognitive deficits in AD patients [20,62]. The specificity of elevated BACE1 levels in AD patient plasma is 88%, suggesting the possibility of BACE1-AS as a potential biomarker for AD [24]. A study published in 2016 by Yao et al., indicates that MALAT1 is downregulated in the CSF of AD patients, emphasizing its potential as a biomarker [2,55]. LncRNA 51A is upregulated in the plasma of AD patients, indicating use as a minimally invasive biomarker for AD [5]. LncRNA BC200 is highly expressed in CNS and has been shown play an important role in neuronal function, synaptogenesis, and immune response [67,68]. In AD brains, the expression levels of lncRNA BC200 are significantly higher when compared with age-matched non-AD brains [69]. Importantly, the extent of increase is associated with the severity of the disease. The plasma levels of lncRNAs BC200 and NEAT1 were found to be significantly higher in late onset AD patients than healthy controls [21], suggesting these lncRNAs can be used for identifying and tracking the progression of late-onset AD. Similarly, a recent study showed altered expression of the lncRNAs TUG1 and FEZF1-AS1 in plasma samples of AD patients [92]. This finding suggests that monitoring plasma lncRNA TUG1and FEZF1-AS1 may serve as valuable biomarkers for AD diagnosis. LncRNAs have emerged as a potential diagnostic tool with predictive and preventive capabilities for multiple described diseases when used as a target or a biomarker. LncRNAs are currently being investigated as biomarkers for lung cancer (NCT 03830619), preeclampsia (NCT 03903393), and ischemic stroke (NCT 04175691) [28]. The levels of various characterized lncRNAs can be measured in blood, urine, epithelia, cerebrospinal fluid (CSF), or other easily obtained diagnostic media to determine the development or progression of certain diseases [1]. LncRNAs possess a far greater potential than is currently being achieved. Therefore, further studies are required to rigorously examine their significance as biomarkers for AD.

## 2. Conclusions and Perspectives

LncRNAs provide a novel avenue of research for the development of new therapies and diagnostic markers for AD and several other human diseases. There is growing consensus that lncRNAs have been implicated in AD pathogenesis, but more research is needed to determine how they influence AD onset and progression. Understanding the regulatory function, interacting partners, and molecular mechanisms of lncRNAs in AD pathophysiology will fuel the development of potential therapeutic targets and identify novel diagnosis biomarkers. Further sights should be set on comprehensive identification and classification of the most practical lncRNAs to target and measure as biomarkers based on their ease of accessibility, abundance, lack of off-target effects, and role(s) in disease pathology. This will include highly sensitive and efficient RNA analysis techniques, advancing bioinformatic technologies and lncRNA databases, and cultivating efficacious lncRNA-mimetics or antagonists. These new findings shift the focus and provide a new lens through which to view the possibilities of novel treatment and diagnostic methods surrounding AD. High-quality RNA therapy does not have off-target or unsought on-target effects but has robust on-target specificity [81]. Understanding the translational challenges of safety, dosage, and delivery is crucial as there is no precedent using lncRNAs in clinical trials. Most notably, dosage will ultimately be determined in human clinical trial as lncRNA therapies may markedly differ from their RNA counterparts. Dosage is contingent on the pharmacokinetics and pharmacodynamic properties and the mechanism of action of the drug [10]. LncRNAs are prime candidates to use as biomarkers and pharmacological targets within neurological disease. Additionally, lncRNAs may provide further insight into the discrepancies between AD incidence and gender. Overall, research on lncRNAs and their connection to neurological disease is growing exponentially, and commercial enterprises are already designing and employing RNA therapeutics. Although much work remains to be done, the path to mechanistic understanding behind neurodegeneration is brighter.

## Figures and Tables

**Table 1:** Examples of lncRNAs implicated in the pathogenesis of AD.

LncRNA ID	Trend	Proposed Function	Reference(s)
MALAT1	↓ in AD	Inhibit neuronal apoptosis & neuroinflammation, neurite outgrowth stimulation	[17,51,52,53,54,55]
NEAT1	↑ in AD	Limits Aβ production and ameliorates cognitive deficits	[21,56,57,58]
MAPT1-AS	↓ in AD	Inhibit tau translation by rRNA pairing	[59,60]
SNHG8	↓ in AD	Interacts with TIA1, an RNA binding protein associated with tau and stress granules	[61]
BACE1-AS	↑ in AD	Amyloid precursor protein cleaving β-secretase → increased Aβ peptides	[18,20,21,62,63]
LRP1-AS	↑ in AD	Lowers LRP1’s expression, influencing Aβ agglomeration	[64]
SORL1-AS	↑ in AD	Creation of deleterious splicing isoforms of SORL1 → increase Aβ levels	[23]
UCHL1-AS	↓ in AD	Ubiquitinates Aβ protein → plaque deconstruction	[24]
51A	↑ in AD	Downregulates SORL1, Impaired processing of APP, increased Aβ formation	[5,35,65,66]
BC200	↑ in AD	Regulating neuronal protein translation → amyloid plaque formation and AD pathogenesis	[21,67,68,69]
NAT-Rad18	↑ in AD	Increases neuronal apoptosis by lowering neuronal DNA damage compensatory abilities	[25,70]
NDM29	↑ in AD	Increased Aβ peptides	[71]
SOX2-OT	↓ in AD	Promotes neurogenesis and neuronal differentiation	[66,72,73]
SNHG1	↑ in AD	Regulate neuroinflammation, DNA methylation and apoptosis	[74,75,76,77]

## Abbreviations

AD: Alzheimer’s disease, Aβ: amyloid beta, ANRIL: antisense noncoding RNA in the INK4 locus, APOE4: apolipoprotein E, APP: Amyloid precursor protein, AS: antisense, BACE1: beta-site amyloid precursor protein cleaving enzyme 1, BBB: blood-brain barrier, circRNAs: Circular RNAs, CNS: central nervous system, CSF: cerebrospinal fluid, EPHB2: ephrin B2 receptor, EOAD: early-onset AD, gRNA: guide RNA, GPCR: G protein-coupled receptor, GDNF: glial cell-derived neurotrophic factor, HMGB2: high-mobility group box 2, KCNA2: Potassium voltage-gated channel subfamily A member 2, lncRNAs: Long non-coding RNAs, LOAD: Late-onset AD, Lrp1: low-density lipoprotein receptor-related protein 1, MALAT1: metastasis-associated lung adenocarcinoma transcript 1, MAPT: microtubule-associated tau protein, MIND: minimally invasive nasal depot, mRNA: messenger RNA, ncRNA: non-coding RNA, NDM29: neuroblastoma differentiation marker 29, NEAT1: Nuclear paraspeckle assembly transcript 1, NFTs: Neurofibrillary tangles, RBPs: RNA-binding proteins, RNA: ribonucleic acid, SNHG1: small nucleolar RNA host gene 1, SORL1: Sortilin-related receptor 1, UCHL1: ubiquitin carboxy-terminal hydrolase L1.

## References

[1] Idda M.L., Munk R., Abdelmohsen K., Gorospe M. (2018). Noncoding RNAs in Alzheimer’s disease. Wiley Interdiscip. Rev. RNA.

[2] Doxtater K., Tripathi M.K., Khan M.M. (2020). Recent advances on the role of long non-coding RNAs in Alzheimer’s disease. Neural Regen. Res..

[3] Riva P., Ratti A., Venturin M. (2016). The Long Non-Coding RNAs in Neurodegenerative Diseases: Novel Mechanisms of Pathogenesis. Curr. Alzheimer Res..

[4] Beam C.R., Kaneshiro C., Jang J.Y., Reynolds C.A., Pedersen N.L., Gatz M. (2018). Differences Between Women and Men in Incidence Rates of Dementia and Alzheimer’s Disease. J. Alzheimers Dis..

[5] Wu X., Xia P., Yang L., Lu C., Lu Z. (2024). The roles of long non-coding RNAs in Alzheimer’s disease diagnosis, treatment, and their involvement in Alzheimer’s disease immune responses. Noncoding RNA Res..

[6] Canoy R.J., Sy J.C., Deguit C.D., Castro C.B., Dimaapi L.J., Panlaqui B.G., Perian W., Yu J., Velasco J.M., Sevilleja J.E. (2024). Non-coding RNAs involved in the molecular pathology of Alzheimer’s disease: A systematic review. Front. Neurosci..

[7] Ilieva M.S. (2024). Non-Coding RNAs in Neurological and Neuropsychiatric Disorders: Unraveling the Hidden Players in Disease Pathogenesis. Cells.

[8] Mattick J.S., Amaral P.P., Carninci P., Carpenter S., Chang H.Y., Chen L.L., Chen R., Dean C., Dinger M.E., Fitzgerald K.A. (2023). Long non-coding RNAs: Definitions, functions, challenges and recommendations. Nat. Rev. Mol. Cell Biol..

[9] Xiong W., Lu L., Li J. (2024). Long non-coding RNAs with essential roles in neurodegenerative disorders. Neural Regen. Res..

[10] Mercer T.R., Munro T., Mattick J.S. (2022). The potential of long noncoding RNA therapies. Trends Pharmacol. Sci..

[11] Marchese F.P., Raimondi I., Huarte M. (2017). The multidimensional mechanisms of long noncoding RNA function. Genome Biol..

[12] Statello L., Guo C.J., Chen L.L., Huarte M. (2021). Gene regulation by long non-coding RNAs and its biological functions. Nat. Rev. Mol. Cell Biol..

[13] Lauretti E., Dabrowski K., Pratico D. (2021). The neurobiology of non-coding RNAs and Alzheimer’s disease pathogenesis: Pathways, mechanisms and translational opportunities. Ageing Res. Rev..

[14] Srinivas T., Mathias C., Oliveira-Mateos C., Guil S. (2023). Roles of lncRNAs in brain development and pathogenesis: Emerging therapeutic opportunities. Mol. Ther..

[15] Zhang Y., Liu H., Niu M., Wang Y., Xu R., Guo Y., Zhang C. (2024). Roles of long noncoding RNAs in human inflammatory diseases. Cell Death Discov..

[16] Faghihi M.A., Modarresi F., Khalil A.M., Wood D.E., Sahagan B.G., Morgan T.E., Finch C.E., St Laurent G., Kenny P.J., Wahlestedt C. (2008). Expression of a noncoding RNA is elevated in Alzheimer’s disease and drives rapid feed-forward regulation of beta-secretase. Nat. Med..

[17] Ma P., Li Y., Zhang W., Fang F., Sun J., Liu M., Li K., Dong L. (2019). Long Non-coding RNA MALAT1 Inhibits Neuron Apoptosis and Neuroinflammation While Stimulates Neurite Outgrowth and Its Correlation With MiR-125b Mediates PTGS2, CDK5 and FOXQ1 in Alzheimer’s Disease. Curr. Alzheimer Res..

[18] Cao M., Li H., Zhao J., Cui J., Hu G. (2019). Identification of age- and gender-associated long noncoding RNAs in the human brain with Alzheimer’s disease. Neurobiol. Aging.

[19] Zhang Z. (2016). Long non-coding RNAs in Alzheimer’s disease. Curr. Top. Med. Chem..

[20] Feng L., Liao Y.T., He J.C., Xie C.L., Chen S.Y., Fan H.H., Su Z.P., Wang Z. (2018). Plasma long non-coding RNA BACE1 as a novel biomarker for diagnosis of Alzheimer disease. BMC Neurol..

[21] Khodayi M., Khalaj-Kondori M., Hoseinpour Feizi M.A., Jabarpour Bonyadi M., Talebi M. (2022). Plasma lncRNA profiling identified BC200 and NEAT1 lncRNAs as potential blood-based biomarkers for late-onset Alzheimer’s disease. EXCLI J..

[22] Nemeth K., Bayraktar R., Ferracin M., Calin G.A. (2024). Non-coding RNAs in disease: From mechanisms to therapeutics. Nat. Rev. Genet..

[23] Pereira Fernandes D., Bitar M., Jacobs F.M.J., Barry G. (2018). Long Non-Coding RNAs in Neuronal Aging. Noncoding RNA.

[24] Wei C.W., Luo T., Zou S.S., Wu A.S. (2018). The Role of Long Noncoding RNAs in Central Nervous System and Neurodegenerative Diseases. Front. Behav. Neurosci..

[25] Quan Z., Zheng D., Qing H. (2017). Regulatory Roles of Long Non-Coding RNAs in the Central Nervous System and Associated Neurodegenerative Diseases. Front. Cell Neurosci..

[26] Begliarzade S., Beilerli A., Sufianov A., Tamrazov R., Kudriashov V., Ilyasova T., Liang Y., Beylerli O. (2023). Long non-coding RNAs as promising biomarkers and therapeutic targets in cervical cancer. Noncoding RNA Res..

[27] Loganathan T., Doss C.G. (2023). Non-coding RNAs in human health and disease: Potential function as biomarkers and therapeutic targets. Funct. Integr. Genom..

[28] Nappi F. (2024). Non-Coding RNA-Targeted Therapy: A State-of-the-Art Review. Int. J. Mol. Sci..

[29] Fardi F., Khasraghi L.B., Shahbakhti N., Salami Naseriyan A., Najafi S., Sanaaee S., Alipourfard I., Zamany M., Karamipour S., Jahani M. (2023). An interplay between non-coding RNAs and gut microbiota in human health. Diabetes Res. Clin. Pract..

[30] Agliano F., Rathinam V.A., Medvedev A.E., Vanaja S.K., Vella A.T. (2019). Long Noncoding RNAs in Host-Pathogen Interactions. Trends Immunol..

[31] Wang J., Zhang Y., Li Q., Zhao J., Yi D., Ding J., Zhao F., Hu S., Zhou J., Deng T. (2019). Influenza Virus Exploits an Interferon-Independent lncRNA to Preserve Viral RNA Synthesis through Stabilizing Viral RNA Polymerase PB1. Cell Rep..

[32] Ding J., Chen J., Yin X., Zhou J. (2023). Current understanding on long non-coding RNAs in immune response to COVID-19. Virus Res..

[33] Shirvani H., Jafari H., Moravveji S.S., Abbasi Faranghizadeh F., Talebi M., Ghanavi J., Esfandi F., Najafi S., Nasiri Moghadam M., Farnia P. (2022). Non-coding RNA in SARS-CoV-2: Progress toward therapeutic significance. Int. J. Biol. Macromol..

[34] Yang B., Xia Z.A., Zhong B., Xiong X., Sheng C., Wang Y., Gong W., Cao Y., Wang Z., Peng W. (2017). Distinct Hippocampal Expression Profiles of Long Non-coding RNAs in an Alzheimer’s Disease Model. Mol. Neurobiol..

[35] Chen L., Guo X., Li Z., He Y. (2019). Relationship between long non-coding RNAs and Alzheimer’s disease: A systematic review. Pathol. Res. Pract..

[36] Mercer T.R., Dinger M.E., Mariani J., Kosik K.S., Mehler M.F., Mattick J.S. (2008). Noncoding RNAs in Long-Term Memory Formation. Neuroscientist.

[37] Wan P., Su W., Zhuo Y. (2017). The Role of Long Noncoding RNAs in Neurodegenerative Diseases. Mol. Neurobiol..

[38] Harrow J., Frankish A., Gonzalez J.M., Tapanari E., Diekhans M., Kokocinski F., Aken B.L., Barrell D., Zadissa A., Searle S. (2012). GENCODE: The reference human genome annotation for The ENCODE Project. Genome Res..

[39] Carlevaro-Fita J., Johnson R. (2019). Global Positioning System: Understanding Long Noncoding RNAs through Subcellular Localization. Mol. Cell.

[40] Quinn J.J., Chang H.Y. (2016). Unique features of long non-coding RNA biogenesis and function. Nat. Rev. Genet..

[41] Bhat S.A., Ahmad S.M., Mumtaz P.T., Malik A.A., Dar M.A., Urwat U., Shah R.A., Ganai N.A. (2016). Long non-coding RNAs: Mechanism of action and functional utility. Noncoding RNA Res..

[42] Oo J.A., Brandes R.P., Leisegang M.S. (2022). Long non-coding RNAs: Novel regulators of cellular physiology and function. Pflug. Arch..

[43] Wang K.C., Chang H.Y. (2011). Molecular mechanisms of long noncoding RNAs. Mol. Cell.

[44] Zhang X., Wang W., Zhu W., Dong J., Cheng Y., Yin Z., Shen F. (2019). Mechanisms and Functions of Long Non-Coding RNAs at Multiple Regulatory Levels. Int. J. Mol. Sci..

[45] Millan M.J. (2017). Linking deregulation of non-coding RNA to the core pathophysiology of Alzheimer’s disease: An integrative review. Prog. Neurobiol..

[46] Li L., Zhuang Y., Zhao X., Li X. (2018). Long Non-coding RNA in Neuronal Development and Neurological Disorders. Front. Genet..

[47] Cuevas-Diaz Duran R., Wei H., Kim D.H., Wu J.Q. (2019). Invited Review: Long non-coding RNAs: Important regulators in the development, function and disorders of the central nervous system. Neuropathol. Appl. Neurobiol..

[48] Lan Z., Chen Y., Jin J., Xu Y., Zhu X. (2021). Long Non-coding RNA: Insight Into Mechanisms of Alzheimer’s Disease. Front. Mol. Neurosci..

[49] Anilkumar A.K., Vij P., Lopez S., Leslie S.M., Doxtater K., Khan M.M., Yallapu M.M., Chauhan S.C., Maestre G.E., Tripathi M.K. (2024). Long Non-Coding RNAs: New Insights in Neurodegenerative Diseases. Int. J. Mol. Sci..

[50] Wang D.Q., Fu P., Yao C., Zhu L.S., Hou T.Y., Chen J.G., Lu Y., Liu D., Zhu L.Q. (2018). Long Non-coding RNAs, Novel Culprits, or Bodyguards in Neurodegenerative Diseases. Mol. Ther. Nucleic Acids.

[51] Bernard D., Prasanth K.V., Tripathi V., Colasse S., Nakamura T., Xuan Z., Zhang M.Q., Sedel F., Jourdren L., Coulpier F. (2010). A long nuclear-retained non-coding RNA regulates synaptogenesis by modulating gene expression. EMBO J..

[52] Chanda K., Jana N.R., Mukhopadhyay D. (2022). Long non-coding RNA MALAT1 protects against Abeta(1-42) induced toxicity by regulating the expression of receptor tyrosine kinase EPHA2 via quenching miR-200a/26a/26b in Alzheimer’s disease. Life Sci..

[53] Chen L., Feng P., Zhu X., He S., Duan J., Zhou D. (2016). Long non-coding RNA Malat1 promotes neurite outgrowth through activation of ERK/MAPK signalling pathway in N2a cells. J. Cell Mol. Med..

[54] Yang W., Zhang S., Li B., Zhang Y. (2018). MALAT1 inhibits proliferation and promotes apoptosis of SH-SY5Y cells induced by Abeta25-35 via blocking PI3K/mTOR/GSK3beta pathway. Xi Bao Yu Fen. Zi Mian Yi Xue Za Zhi.

[55] Yao J., Wang X.Q., Li Y.J., Shan K., Yang H., Wang Y.N., Yao M.D., Liu C., Li X.M., Shen Y. (2016). Long non-coding RNA MALAT1 regulates retinal neurodegeneration through CREB signaling. EMBO Mol. Med..

[56] Li K., Wang Z. (2023). lncRNA NEAT1: Key player in neurodegenerative diseases. Ageing Res. Rev..

[57] Ke S., Yang Z., Yang F., Wang X., Tan J., Liao B. (2019). Long Noncoding RNA NEAT1 Aggravates Abeta-Induced Neuronal Damage by Targeting miR-107 in Alzheimer’s Disease. Yonsei Med. J..

[58] Huang Z., Zhao J., Wang W., Zhou J., Zhang J. (2020). Depletion of LncRNA NEAT1 Rescues Mitochondrial Dysfunction Through NEDD4L-Dependent PINK1 Degradation in Animal Models of Alzheimer’s Disease. Front. Cell Neurosci..

[59] Rogers B.B., Anderson A.G., Lauzon S.N., Davis M.N., Hauser R.M., Roberts S.C., Rodriguez-Nunez I., Trausch-Lowther K., Barinaga E.A., Hall P.I. (2024). Neuronal MAPT expression is mediated by long-range interactions with cis-regulatory elements. Am. J. Hum. Genet..

[60] Simone R., Javad F., Emmett W., Wilkins O.G., Almeida F.L., Barahona-Torres N., Zareba-Paslawska J., Ehteramyan M., Zuccotti P., Modelska A. (2021). MIR-NATs repress MAPT translation and aid proteostasis in neurodegeneration. Nature.

[61] Bhagat R., Minaya M.A., Renganathan A., Mehra M., Marsh J., Martinez R., Eteleeb A.M., Nana A.L., Spina S., Seeley W.W. (2023). Long non-coding RNA SNHG8 drives stress granule formation in tauopathies. Mol. Psychiatry.

[62] Fotuhi S.N., Khalaj-Kondori M., Hoseinpour Feizi M.A., Talebi M. (2019). Long Non-coding RNA BACE1-AS May Serve as an Alzheimer’s Disease Blood-Based Biomarker. J. Mol. Neurosci..

[63] Zhang W., Zhao H., Wu Q., Xu W., Xia M. (2018). Knockdown of BACE1-AS by siRNA improves memory and learning behaviors in Alzheimer’s disease animal model. Exp. Ther. Med..

[64] Yamanaka Y., Faghihi M.A., Magistri M., Alvarez-Garcia O., Lotz M., Wahlestedt C. (2015). Antisense RNA controls LRP1 Sense transcript expression through interaction with a chromatin-associated protein, HMGB2. Cell Rep..

[65] Ciarlo E., Massone S., Penna I., Nizzari M., Gigoni A., Dieci G., Russo C., Florio T., Cancedda R., Pagano A. (2013). An intronic ncRNA-dependent regulation of SORL1 expression affecting Abeta formation is upregulated in post-mortem Alzheimer’s disease brain samples. Dis. Model. Mech..

[66] Asadi M.R., Hassani M., Kiani S., Sabaie H., Moslehian M.S., Kazemi M., Ghafouri-Fard S., Taheri M., Rezazadeh M. (2021). The Perspective of Dysregulated LncRNAs in Alzheimer’s Disease: A Systematic Scoping Review. Front. Aging Neurosci..

[67] Li H., Zheng L., Jiang A., Mo Y., Gong Q. (2018). Identification of the biological affection of long noncoding RNA BC200 in Alzheimer’s disease. Neuroreport.

[68] Liu N.X., Li Q.H. (2020). LncRNA BC200 regulates neuron apoptosis and neuroinflammation via PI3K/AKT pathway in Alzheimer’s disease. J. Biol. Regul. Homeost. Agents.

[69] Mus E., Hof P.R., Tiedge H. (2007). Dendritic BC200 RNA in aging and in Alzheimer’s disease. Proc. Natl. Acad. Sci. USA.

[70] Parenti R., Paratore S., Torrisi A., Cavallaro S. (2007). A natural antisense transcript against Rad18, specifically expressed in neurons and upregulated during beta-amyloid-induced apoptosis. Eur. J. Neurosci..

[71] Massone S., Ciarlo E., Vella S., Nizzari M., Florio T., Russo C., Cancedda R., Pagano A. (2012). NDM29, a RNA polymerase III-dependent non coding RNA, promotes amyloidogenic processing of APP and amyloid beta secretion. Biochim. Biophys. Acta.

[72] Knauss J.L., Miao N., Kim S.N., Nie Y., Shi Y., Wu T., Pinto H.B., Donohoe M.E., Sun T. (2018). Long noncoding RNA Sox2ot and transcription factor YY1 co-regulate the differentiation of cortical neural progenitors by repressing Sox2. Cell Death Dis..

[73] Arisi I., D’Onofrio M., Brandi R., Felsani A., Capsoni S., Drovandi G., Felici G., Weitschek E., Bertolazzi P., Cattaneo A. (2011). Gene expression biomarkers in the brain of a mouse model for Alzheimer’s disease: Mining of microarray data by logic classification and feature selection. J. Alzheimers Dis..

[74] Chen H., Zhang C.J., Zhao Z.Y., Gao Y.Y., Zhao J.T., Li X.X., Zhang M., Wang H. (2024). Mechanisms underlying LncRNA SNHG1 regulation of Alzheimer’s disease involve DNA methylation. J. Toxicol. Environ. Health Part A.

[75] Cao B., Wang T., Qu Q., Kang T., Yang Q. (2018). Long Noncoding RNA SNHG1 Promotes Neuroinflammation in Parkinson’s Disease via Regulating miR-7/NLRP3 Pathway. Neuroscience.

[76] Zhang M., Zhang Y.Q., Wei X.Z., Lee C., Huo D.S., Wang H., Zhao Z.Y. (2019). Differentially expressed long-chain noncoding RNAs in human neuroblastoma cell line (SH-SY5Y): Alzheimer’s disease cell model. J. Toxicol. Environ. Health Part A.

[77] Gao Y., Zhang N., Lv C., Li N., Li X., Li W. (2020). lncRNA SNHG1 Knockdown Alleviates Amyloid-beta-Induced Neuronal Injury by Regulating ZNF217 via Sponging miR-361-3p in Alzheimer’s Disease. J. Alzheimers Dis..

[78] Ding Y., Luan W., Shen X., Wang Z., Cao Y. (2022). LncRNA BDNF-AS as ceRNA regulates the miR-9-5p/BACE1 pathway affecting neurotoxicity in Alzheimer’s disease. Arch. Gerontol. Geriatr..

[79] Massone S., Vassallo I., Fiorino G., Castelnuovo M., Barbieri F., Borghi R., Tabaton M., Robello M., Gatta E., Russo C. (2011). 17A, a novel non-coding RNA, regulates GABA B alternative splicing and signaling in response to inflammatory stimuli and in Alzheimer disease. Neurobiol. Dis..

[80] Yue D., Guanqun G., Jingxin L., Sen S., Shuang L., Yan S., Minxue Z., Ping Y., Chong L., Zhuobo Z. (2020). Silencing of long noncoding RNA XIST attenuated Alzheimer’s disease-related BACE1 alteration through miR-124. Cell Biol. Int..

[81] Winkle M., El-Daly S.M., Fabbri M., Calin G.A. (2021). Noncoding RNA therapeutics—Challenges and potential solutions. Nat. Rev. Drug Discov..

[82] Qureshi I.A., Mehler M.F. (2013). Long non-coding RNAs: Novel targets for nervous system disease diagnosis and therapy. Neurotherapeutics.

[83] Wang X., Zhang M., Liu H. (2019). LncRNA17A regulates autophagy and apoptosis of SH-SY5Y cell line as an in vitro model for Alzheimer’s disease. Biosci. Biotechnol. Biochem..

[84] Lin P., Wang J., Li Y., Li G., Wang Y. (2024). LINC00472 Regulates Ferroptosis of Neurons in Alzheimer’s Disease via FOXO1. Dement. Geriatr. Cogn. Disord..

[85] Balusu S., Horre K., Thrupp N., Craessaerts K., Snellinx A., Serneels L., T’Syen D., Chrysidou I., Arranz A.M., Sierksma A. (2023). MEG3 activates necroptosis in human neuron xenografts modeling Alzheimer’s disease. Science.

[86] Shou F., Li G., Morshedi M. (2024). Long Non-coding RNA ANRIL and Its Role in the Development of Age-Related Diseases. Mol. Neurobiol..

[87] Ghafouri-Fard S., Safari M., Taheri M., Samadian M. (2022). Expression of Linear and Circular lncRNAs in Alzheimer’s Disease. J. Mol. Neurosci..

[88] Zhou B., Li L., Qiu X., Wu J., Xu L., Shao W. (2020). Long non-coding RNA ANRIL knockdown suppresses apoptosis and pro-inflammatory cytokines while enhancing neurite outgrowth via binding microRNA-125a in a cellular model of Alzheimer’s disease. Mol. Med. Rep..

[89] Guo L., Zhong M.B., Zhang L., Zhang B., Cai D. (2022). Sex Differences in Alzheimer’s Disease: Insights From the Multiomics Landscape. Biol. Psychiatry.

[90] Shi C., Zhang L., Qin C. (2017). Long non-coding RNAs in brain development, synaptic biology, and Alzheimer’s disease. Brain Res. Bull..

[91] Jones C.H., Androsavich J.R., So N., Jenkins M.P., MacCormack D., Prigodich A., Welch V., True J.M., Dolsten M. (2024). Breaking the mold with RNA-a “RNAissance” of life science. npj Genom. Med..

[92] Dibaj M., Haghi M., Safaralizadeh R., Saberi A. (2024). The role of EZH2 and its regulatory lncRNAs as a serum-based biomarker in Alzheimer’s disease. Mol. Biol. Rep..

